# Land tenure shapes black bear density and abundance on a multi‐use landscape

**DOI:** 10.1002/ece3.4617

**Published:** 2018-12-18

**Authors:** Anne E. Loosen, Andrea T. Morehouse, Mark S. Boyce

**Affiliations:** ^1^ Department of Biological Sciences University of Alberta Edmonton Alberta Canada

**Keywords:** American black bear, habitat, hunting, population estimation, resource‐selection function, spatially explicit capture–recapture, *Ursus americanus*

## Abstract

Global biodiversity is decreasing rapidly. Parks and protected lands, while designed to conserve wildlife, often cannot provide the habitat protection needed for wide‐ranging animals such as the American black bear (*Ursus americanus*). Conversely, private lands are often working landscapes (e.g., farming) that have high human footprints relative to protected lands. In southwestern Alberta, road densities are highest on private lands and black bears can be hunted year‐round. On protected lands, road densities are lowest, and hunting is prohibited. On public lands under the jurisdiction of the provincial government (Crown lands), seasonal hunting is permitted. Population estimates are needed to calculate sustainable harvest levels and to monitor population trends. In our study area, there has never been a robust estimate of black bear density and spatial drivers of black bear density are poorly understood. We used non‐invasive genetic sampling and indices of habitat productivity and human disturbance to estimate density and abundance for male and female black bears in 2013 and 2014 using two methods: spatially explicit capture–recapture (SECR) and resource‐selection functions (RSF). Land tenure best explained spatial variation in black bear density. Black bear densities for females and males were highest on parkland and lowest on Crown lands. Sex ratios were female‐biased on private lands, likely a result of lower harvests and movement of females out of areas with high male density. *Synthesis and application*: Both SECR and RSF methods clearly indicate spatial structuring of black bear density, with a strong influence based on how lands are managed. Land tenure influences the distribution of available foods and risk from humans. We emphasize the need for improved harvest reporting, particularly for non‐licensed hunting on private land, to estimate the extent of black bear harvest mortality.

## 1 INTRODUCTION

Increases in the global human population and associated infrastructure development are increasing habitat fragmentation and destruction, and global biodiversity is rapidly decreasing (Benítez‐López, Alkemade, & Verweij, 2010). Parks and protected areas often serve as areas of reduced human influence to conserve and protect habitats. While many wildlife species have done well within this framework (e.g., grizzly bear [*Ursus arctos*] recovery in Yellowstone National Park; van Manen et al., 2016), parks and protected areas have been criticized for preserving scenic beauty rather than biodiversity or connectivity (Jenkins, Van Houtan, Pimm, & Sexton, 2015). In North America, mountainous protected areas have a high proportion of rock and ice, which for many species does not provide adequate foraging opportunities (Joppa & Pfaff, 2009). Further, these areas are commonly of low soil fertility, which, in turn, can result in nutrient‐poor areas and increased chances of food shortages (Rogers, 1987). For wide‐ranging species, small protected areas do not encompass year‐round habitat and the local extinction rate of mammals has been found to be inversely related to spatial extent of the protected area (Newmark, 1995).

Even where protected areas exist, wildlife commonly will use adjacent, unprotected private and public lands for dispersal, migration, foraging, breeding, and overwintering (Berger, 2004). Often, unprotected lands are working landscapes, such as agricultural lands used for farming and ranching. These lands can be productive and attractive to animals for their high‐quality forage (Sayre, Carlisle, Huntsinger, Fisher, & Shattuck, 2012), as well as provide food subsidies from agriculture such as stored and standing grain and hay, silage, livestock, dead livestock, and beeyards (Wilson, Madel, Mattson, Graham, & Merrill, 2006). In southwestern Alberta, Canada, private lands are primarily agricultural areas used for cattle ranching, cereal grain, and oilseed farming. While private lands in this area have been shown to be attractive to grizzly bears (Northrup, Stenhouse, & Boyce, 2012), little is known about what drives spatial variation for other large carnivores, including black bears (*Ursus americanus*).

In North America, black bears are legally harvested throughout much of their range (Garshelis, 1990). In hunted populations, abundance and density estimates are needed to calculate sustainable harvest levels (Williams, Nichols, & Conroy, 2002). Despite low power to detect population trends, harvest data often are the only information biologists have to assess trends or to set harvest objectives (Garshelis & Hristienko, 2006). In Alberta, hunters are allowed to harvest 12% of the estimated provincial population, but the most recent population estimates are 20–30 years old (Gunson & Markham, 1993). In southwestern Alberta, the provincial government derived the minimum number of black bears in permanently occupied habitats by using percent cover of habitat, with minimum black bear densities for an ecoregion extrapolated from an adjacent study area and wildlife management unit (Gunson & Markham, 1993). Using this method, minimum black bear density was estimated to be 52.9 bears/1,000 km^2^ (excluding Waterton Lakes National Park [WLNP]), which was low relative to other wildlife management units in Alberta (Gunson & Markham, 1993). Further, no error estimates were calculated so we do not know the extent of variance around the mean. Thus, there is a need to improve both the empirical data and the sophistication of the methods used to estimate density.

Spatially explicit capture–recapture and resource‐selection functions (RSF) are two methods that can be used to estimate population density and account for spatiotemporal factors affecting density. SECR models use location information from detection events to estimate the distribution of animal home‐range centers and to account for the effects of animal space use and home‐range centers on the detection process (Efford, 2004; Royle, Chandler, Sollmann, & Gardner, 2014). These models require large amounts of field data (Czaplewski, Crowe, & McDonald, 1983), which can be time and cost intensive to collect (Royle & Nichols, 2003), and must meet multiple assumptions to avoid biasing parameter estimates and model over‐parameterization (Choquet, Lebreton, Gimenez, Reboulet, & Pradel, 2009; Fletcher et al., 2012).

Animal densities are usually related to habitat selection (Boyce et al., 2016), and this relationship has been explored using RSFs (Johnson, Nielsen, Merrill, McDonald, & Boyce, 2006). For example, Boyce and McDonald (1999) associated abundance with RSF scores, which could then be extrapolated to new or unsampled areas. To date, RSF‐abundance extrapolations have been applied primarily to theoretical or expanding populations (Boyce & Waller, 2003; Mladenoff & Sickley, 1998). Projections are important to anticipate how habitats could shape population expansions. Because the RSF‐abundance extrapolation only requires presence data, it could provide a low‐cost alternative to estimating density and abundance for wildlife managers, who often operate on restricted budgets. However, this method has never been applied to a population with concurrent mark–recapture data.

We use SECR models to estimate black bear density from DNA data at rub trees and compare these to RSF‐based results. Using non‐invasive genetic sampling (NGS) data collected from rub trees, power poles, fence posts, and fence lines (hereafter, rub objects), our objectives were to (a) estimate black bear density using both SECR and RSF methods for the same data, and (b) use information‐theoretic methods with SECR and RSF to identify spatial covariates that best explain spatial variation in density. We used black bear NGS data from southwestern Alberta (2013–2014), habitat and human‐disturbance covariates, and grizzly bear detection data to account for spatial variation in black bear density. We predicted black bear densities would be highest in protected areas where road densities are lowest hunting is prohibited, and where forested escape terrain from grizzly bears is more abundant. Black bear populations can be female‐biased in un‐hunted populations. This bias is exacerbated in hunted populations, however, because hunters disproportionately select males (Bunnell & Tait, 1980; Miller, 1990). We predicted higher female densities relative to males.

## 2 STUDY AREA

**Figure 1:**
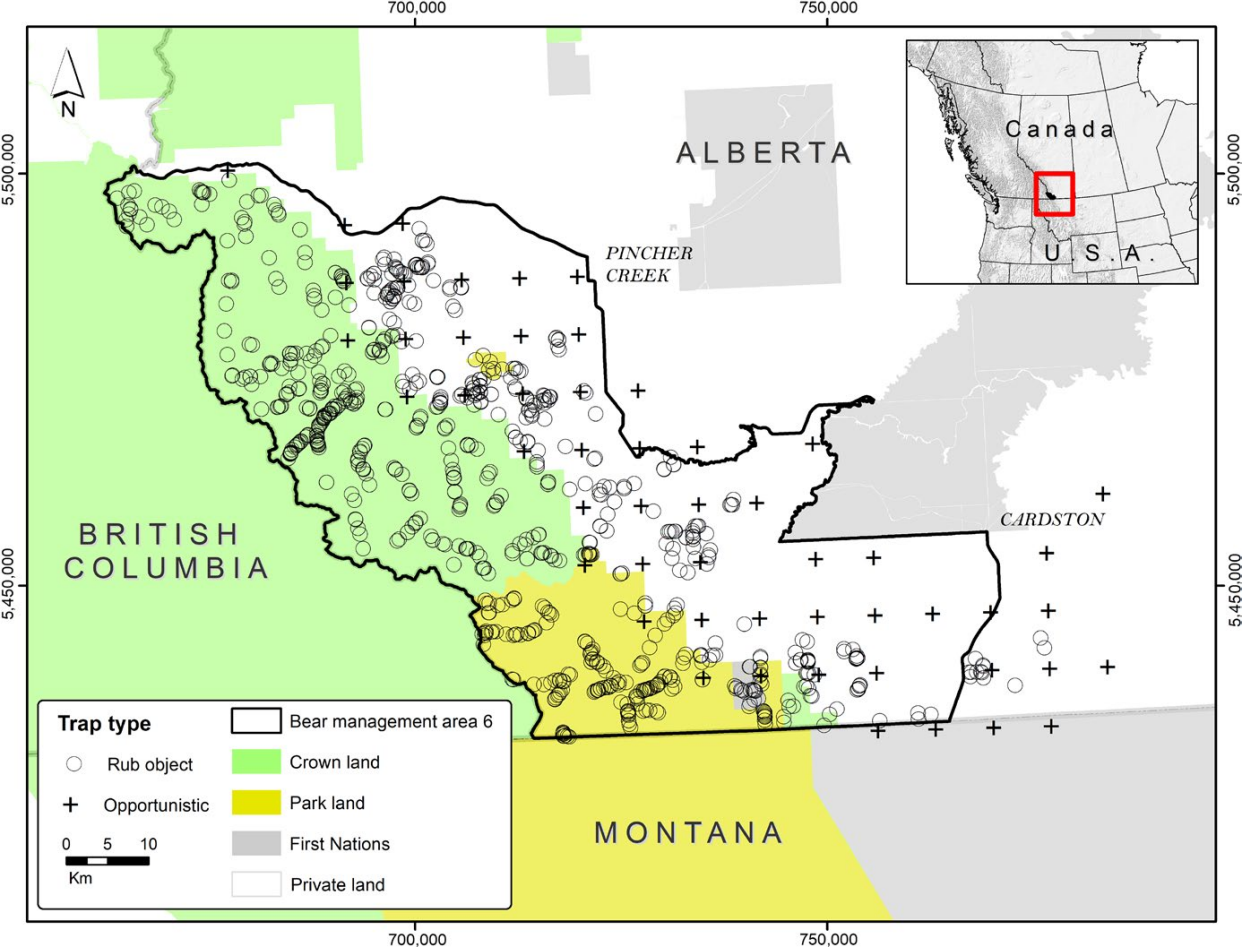
Black bear hair samples were collected in 2013 and 2014 in southwestern Alberta, Canada. Grid centroids (cross) represent opportunistic surveying by landowners, Fish and Wildlife Officers, and project technicians. Our study area roughly aligns with provincial bear management area 6

The 3,600 km^2^ study area is in the southern Canadian Rocky Mountains and is bounded by Highway 3 to the north, British Columbia to the west, the United States–Canada border to the south, and Highway 2 to the east (Figure 1). The area includes WLNP, which borders Glacier National Park (GNP), USA. The area is a mix of land‐cover types: conifer forest (29%), agricultural (22%), native grassland and cultivated fields (16%), shrubland (16%), and deciduous forest (11%). Agriculture is the primary industry (Statistics Canada, MD of Pincher Creek, 2011 Community Profile).

Land management in southwestern Alberta is varied. Private land (1,872 km^2^; 52% of area) has the highest road density (1.3 km/km^2^; Northrup et al., 2012) and is characterized by rough fescue grasslands and agriculture. Crown lands (i.e., public lands under the jurisdiction of the provincial government; 1,204 km^2^; 34% of area) have lower road density (0.55 km/km^2^) relative to private land, and licensed black bear hunting occurs in the spring (1 April–31 May) and fall (1 September–30 November). Crown lands are characterized by alpine, montane, and aspen‐parkland habitat (Northrup et al., 2012). Protected areas (511 km^2^; 14% of area), which includes WLNP and Beauvais Provincial Park, has the lowest road density (0.18 km/km^2^), and hunting is prohibited. These areas are characterized by alpine, montane, and aspen‐parkland vegetation.

## 3 METHODS

### 3.1 Hair collection and analysis

In 2011 and 2012, we surveyed for and set up rub objects (Figure 1). We searched for rub objects based on bear travel corridors, roads and trails, local knowledge, and grizzly bear resource‐selection maps (Northrup et al., 2012). We established rub objects where we observed fresh hair and attached four barbed‐wire segments to each object. We also surveyed barbed‐wire fencelines for hair and marked start and end points for resurvey.

By 2013, we established 873 rub objects. All rub objects were visited once every 21 days from May to November in 2013 and 2014 (Table 1). We removed all hair during the first visit of each year. The remaining seven visits were collection events, and we collected all hair that was present. We considered a barb or end of a wire a discrete sampling unit, and thus, hair was collected only from the wire. After collection, we burned remnant hairs to prevent false recaptures. A second data source included “opportunistic” hair samples collected by landowners, Fish and Wildlife officers visiting conflict sites, and field technicians. Opportunistic samples were collected throughout bears’ active months (March–December) and were assigned to an eighth sample occasion in 2013 and 2014, as is common with opportunistic samples in traditional mark–recapture (Kendall et al., 2009, 2015).

**Table 1:** Black bear detections from non‐invasive genetic sampling in southwestern Alberta, Canada

Year	Occasion	Number of active rub objects^a^	Number of rub objects detecting a black bear		Hair collection dates	Number of new black bears		Number of individuals detected		Number of detections	
			M	F		M	F	M	F	M	F
2013	1	808	96	16	June 17–July 7	56	15	56	15	99	16
	2	816	78	25	July 8–July 28	25	21	53	23	78	26
	3	809	44	24	July 29–August 18	11	17	29	21	46	24
	4	828	13	39	August 19–September 8	8	25	13	35	16	41
	5	836	20	22	September 9–September 29	9	8	15	19	20	22
	6	846	22	29	September 30–October 20	10	7	22	23	23	29
	7	777	12	10	October 21–November 8	5	3	10	10	12	11
	8	48	8	8	Apr 30–October 31	2	5	12	8	12	8
		Total	293	173		126	101			306	177
2014	1	861	103	25	June 17–July 6	62	22	62	22	108	27
	2	871	75	26	July 7–July 27	25	19	49	24	77	27
	3	869	32	19	July 28–August 17	10	12	30	18	34	20
	4	869	19	23	August 18–September 7	4	12	15	18	19	23
	5	870	16	32	September 8–September 28	7	19	15	26	16	32
	6	872	18	16	September 29–October 19	8	4	14	13	18	16
	7	867	7	10	October 20–November 9	4	4	7	8	7	10
	8	54	12	11	May 20–October 14	2	8	13	11	15	13
		Total	282	162		122	100			294	168

Notes

Data were collected from rub objects (*n* = 873) in 2013 and 2014. Data from opportunistic samples were grouped into the eighth occasion.

a Number of active sampling stations may vary depending on destruction of a rub tree from windfall or avalanche, access issues because of heavy snow, flooding, or discovery and set up of new rub trees.

Hair samples were stored in coin envelopes and sent to Wildlife Genetics International (WGI; Nelson, British Columbia) for genetic analysis to determine species and individual via multi‐locus microsatellite analysis of nuclear DNA (Paetkau, 2003, 2004). Sex was assigned using the amelogenin marker (Ennis & Gallagher, 1994). In the Rocky Mountains, typically only one individual is identified during a specific rub/date combination (Sawaya, Stetz, Clevenger, Gibeau, & Kalinowski, 2012; Stetz, Kendall, & Macleod, 2014). Morehouse and Boyce (2016) explored grizzly bear data from southwestern Alberta using various subsampling strategies and found that subsampling every third hair sample in the genetics laboratory maximized detections of individuals while remaining cost‐effective. We used the same approach here. All hair samples were subjected to a three‐phase process of first pass, cleanup, and error‐check (Paetkau, 2003, 2004) to establish an eight‐locus marker system common to the Rocky Mountains (Paetkau, Calvert, Stirling, & Strobeck, 1995; Sawaya et al., 2012).

### 3.2 Spatially explicit capture–recapture

Spatially explicit capture–recapture assumes the probability of detection is a decreasing function of the distance between an animal's home‐range center and the rub object and parameterizes the following: *g*_0_ is the probability of detection if the trap is located at the animal's home‐range center; sigma (*σ*) is a parameter that describes the spatial scale over which capture probability declines (Efford, 2004). Instead of *g*_0_, we used lambda_0_ (*λ*_0_), the cumulative hazard of detection (expected number of detections per unit time at a location and time) which has a more direct relationship with home‐range activity than probability. The equation relating *λ*_0_ and *g*_0_ is *g*(*d*) = 1 − exp(−*λ*(*d*)) where *g* is the probability of detection and *d* is the distance between trap location and an animal's home‐range center (Efford, 2004; Efford, Borchers, & Byrom, 2009). We used a binomial distribution and a hazard half‐normal function with a full likelihood to estimate density (*D*), *σ*, and *λ*_0_ (Efford, 2004). All analyses were run in program R v.3.2.1 (R Development Core Team 2015) using package “secr” (Efford, 2016).

The area of integration sets the outer spatial limits for which home‐range centers can be assigned and should encompass all individuals that could have been exposed to the trap array (Efford & Fewster, 2013; Efford & Mowat, 2014). For both males and females, we calculated the area of integration using three times the root pooled spatial variance (RPSV), which is a 2D measure of dispersion of detections around trap locations (Efford, 2016). We calculated RPSV for each sex and added the largest value for each sex (18 km for males, 13 km for females) as a buffer around the study area. “Secr” models discretize continuous habitat by using a gridded mask, on which we built spatial models of density. We used a mask with spacing of 2.5 km between grid centroids. We completed a sensitivity analysis, which suggested that our area of integration and spacing were a good compromise between processing time and minimizing bias (minimal change in *D* or log‐likelihood).

Each year, we associated each opportunistic sample with a 7 × 7‐km cell centroid (Sawaya et al., 2012; Stetz et al., 2014). Like unstructured scat dog searches with non‐fixed trap locations, we defined the spatial extent of the grid based on locations searched by technicians, landowners, and Fish and Wildlife Officers (Thompson, Royle, & Garner, 2012). Each cell then became a trap location for opportunistic samples, allowing for both “0” and “1” data necessary for mark‐recapture. Because we could not quantify search effort for opportunistic samples, we assumed a uniform observation process for encountering hair samples within each grid cell. We believe this to be justifiable because increased effort affects precision, but not accuracy of SECR estimates (Mollet, Kéry, Gardner, Pasinelli, & Royle, 2015; Morehouse & Boyce, 2016).

Because “secr” models are computationally intensive, we designed a 2‐step modeling approach (Table 2). In step 1, we identified the most parsimonious observation model (i.e., *σ* and *λ*_0_) while holding density constant (*D* ~ 1). In step 2, we used the most parsimonious observation model as a base model on which to build a full model (i.e., *D*,* σ*, and *λ*_0_; Table 2). For step 1, we created 17 single‐session models for each sex in each year that differed in factors affecting *σ* and *λ*_0_ (Table 2). While rub trees offer no lure or bait, there is a potential for individual rub objects to be favored and for individual bears to exhibit variation in rubbing behavior. If rubbing is related to dominance or breeding (Clapham, Nevin, Ramsey, & Rosell, 2012; Lamb et al., 2017), rubbing may be influenced by other bears. The trap‐specific behavioral response (bk) allows for a step change after first detection of an individual at a site. We used trap type as a covariate for both *σ* and *λ*_0_. We assumed variation in cumulative hazard rates of detection among trap types (*rub*: trees, power poles, fenceposts, *fence*: fencelines, and *opp*: opportunistic). Because bears interact with fencelines and rub trees differently, we would expect to see differential space use between trap types (i.e., a sampling effect).

**Table 2:** Step 1 and 2 candidate SECR models for black bears in southwestern Alberta. We used a hazard half‐normal detection function for all models

Step number	Model number	Model description
1	1	*D* ~ 1 *λ*_0_ * *~ 1 *σ* ~ 1
	2	*D *~ 1 *λ*_0_ * *~ bk *σ* ~ 1
	3	*D *~ 1 *λ*_0_ * *~ traptype *σ* ~ 1
	4	*D *~ 1 *λ*_0_ * *~ 1 *σ* ~ traptype
	5	*D *~ 1 *λ*_0_ * *~ traptype *σ* ~ traptype
	6	*D *~ 1 *λ*_0_ * *~ *T σ* ~ 1
	7	*D *~ 1 *λ*_0_ * *~ *T σ* ~ *T*
	8	*D *~ 1 *λ*_0_ * *~ 1 *σ* ~ *T*
	9	*D *~ 1 *λ*_0_ * *~ traptype + bk *σ* ~ traptype
	10	*D *~ 1 *λ*_0_ * *~ traptype + bk *σ* ~ 1
	11	*D *~ 1 *λ*_0_ * *~ traptype + bk *σ* ~ *T*
	12	*D *~ 1 *λ*_0_ * *~ GB + *T σ *~ 1
	13	*D *~ 1 *λ*_0_ * *~ GB + *T* + traptype *σ *~ 1
	14	*D *~ 1 *λ*_0_ * *~ *T* + cover *σ *~ GB
	15	*D *~ 1 *λ*_0_ * *~ traptype + bk *σ *~ GB
	16	*D *~ 1 *λ*_0_ * *~ *T* + cover + bk *σ *~ 1
	17	*D *~ 1 *λ*_0_ * *~ traptype + cover + bk *σ *~ 1
2	1	*D *~ burn + base model from step 1
	2	*D *~ tenure + base model from step 1
	3	*D *~ harvest + base model from step 1
	4	*D *~ tertiary rd + base model from step 1
	5	*D *~ canopy + base model from step 1
	6	*D *~ water + base model from step 1
	7	*D *~ ndvi + base model from step 1
	8	*D *~ rddens + base model from step 1

GB: grizzly bear; SECR: spatially explicit capture–recapture.

Model parameters include density (*D*), *λ*_0_, and *σ*. *λ*_0_ is the cumulative hazard of detection, and *σ* is the spatial scale parameter. *D *~ 1 indicates homogenous density. See Section 2 for covariate definitions

A bear's decision to rub could be influenced by the bear that rubbed previously (Clapham et al., 2012; Lamb et al., 2017). Because grizzly bears are dominant over black bears (Herrero, 1978) and can be displaced via interspecific competition (Holm, Lindzey, & Moody, 1999), we created a time‐varying index of grizzly bear detection (GB; 1 = detected during previous occasion, 0 = not detected during previous occasion) at each rub object for each sampling occasion (grizzly bear data from Morehouse & Boyce, 2016). Bear use of rub objects varies seasonally (Kendall et al., 2008), which can influence detection probabilities. We included time trend (*T*) as a covariate for *σ* and *λ*_0_. Last, tree cover provides security to black bears and cover type could influence detection. We included a singular habitat covariate with seven levels: deciduous, coniferous, shrub, grassland, agriculture, mixed forest, barren (30‐m resolution; Crown Managers Partnership). We would expect deciduous and coniferous cover to positively influence detection.

In step 2, we wanted to identify spatial drivers of black bear density. We created eight a priori single‐session models for each sex in each year (Table 2). Our objective was to identify variables that best explained black bear density, not all possible covariates. As well, “secr” models are time intensive to fit and additional density covariates can make model fitting a challenge. We intentionally kept our density covariates simple. Density covariates included recently burned areas (burn; <20‐year‐old; 30‐m resolution), canopy cover (canopy; 0%–100%; 30‐m resolution), land tenure (tenure; Crown, private, protected), ln‐transformed distance to nearest lakes and major streams (water; 30‐m resolution), ln‐transformed distance to nearest tertiary roads (tertiary rd; 30‐m resolution), road density (km/km^2^; 30‐m resolution) using a 7‐km search radius, Normalized Difference Vegetation Index (NDVI), and hunter harvest.

Normalized Difference Vegetation Index has been correlated with net primary productivity, leaf area index, carbon assimilation, and evapotranspiration (Pettorelli et al., 2011), and has been associated with grizzly bear habitat selection (Northrup et al., 2012). Bare soil, clouds, and concrete correspond to low or zero NDVI values, water has negative values, and green areas correspond to high NDVI values. In our area, high values of NDVI correspond to conifer and aspen forests. We included mean annual MODIS NDVI data from June to November as an indicator of vegetation greenness, or vegetation quality (250‐m resolution; Pettorelli et al., 2011). Last, we estimated hunter harvest for British Columbia, Montana, and Alberta. Montana requires reporting of harvested black bears, whereas Alberta and British Columbia’s harvest data are acquired from annual volunteer hunter harvest surveys. We defined harvest density as the reported number of bears harvested the year prior to sampling, divided by the respective wildlife mangement unit area (km^2^).

For all density covariates, we used the “addCovariates” function, which is a spatial point extraction using the x–y coordinates of the mask grid (Efford, 2016). We used Akaike information criterion corrected for small sample sizes (AICc) to identify the most parsimonious models (Burnham & Anderson, 2002).

Our sampling period extended from June to early November, which is long relative to other NGS bear studies (Kendall et al., 2008). SECR models assume stationarity in home‐range centers and demographic closure, and we would likely be violating this assumption because black bears will increase movements in the fall to acquire enough food resources for winter dormancy. However, SECR models are robust to the closure violation (Efford & Fewster, 2013; Obbard, Howe, & Kyle, 2010). Efford and Mowat (2014) described an inverse and compensatory relationship between *σ* and *λ*_0_ (or *g*_0_), with negligible effect on density. During exploratory modeling, we ran early‐season only and full‐season models using our grizzly bear data (Morehouse & Boyce, 2016), and while *σ* and *λ*_0_ changed, density did not.

### 3.3 Resource‐selection functions

To estimate density using RSFs, we (a) defined the area of inference; (b) calculated RSFs for male and female black bears in WLNP, the reference area; (c) associated abundance (N) with habitat selection in the reference area; (d) extrapolated N across the remaining area of inference and calculated density.

We anticipated that rub object locations were biased because surveys were primarily limited to linear features. To quantify this bias, we compared habitat covariates associated with all rub object locations to random locations. We defined the study area as a minimum convex polygon (MCP) bounding all unique rub object locations (*n *=* *873). We buffered the MCP by 2.4 km, so random points could be in all cardinal directions from rub objects. Our buffer distance represents the average daily linear movement of grizzly bears in the neighboring Flathead Valley, British Columbia (Apps, McLellan, & Woods, 2006), which we would expect to be the maximum daily linear movements of black bears in our study area. We generated 17,460 random points (20:1 random:used) within the MCP and used an exponential RSF, fitted using logistic regression:

$$\text{RSF}\left(x\right)=\exp\left(\beta_{1}x_{1}+\beta_{2}x_{2}+\beta_{3}x_{3}+\ldots +\beta_{n}x_{n}\right),$$

where *β*_*i*_ represents the selection coefficient for covariate *x*_*i*_ in a vector, ***x***, of *n* covariates (Johnson et al., 2006; Manly, McDonald, Thomas, McDonald, & Erickson, 2002). We used a global model that included all density covariates described for “secr” models, as well as ln‐transformed distance to nearest building (house; 30‐m resolution), ln‐transformed distance to nearest secondary road (secondary rd; 30‐m resolution), ln‐transformed distance to nearest primary road (primary rd; 30‐m resolution), terrain ruggedness (TRI; 30‐m resolution), and elevation (30‐m resolution). We standardized all continuous predictor covariates to have $\overset{¯}{x}=0$ and *SD* = 1. From the global model, we created a raster layer (250‐m resolution) in ArcMap (v. 10.3.1; Environmental Systems Research Institute, Redlands, CA, USA). We reclassified RSF values into 10 groups.

Second, we used black bear detection locations to create separate RSFs for male and female black bears. We compared locations where we detected each sex (used) anytime in 2013 and 2014, to the full set of unique rub objects (available). Although these data were derived from the same dataset used for “secr,” RSF data are simple presence/available data, whereas time‐varying data were used for “secr.” Thus, “secr” and RSF datasets are structured differently, making this exercise possible. We used the same covariates described for the global RSF, as well as grizzly bear use (GBU) which is the sum of unique grizzly bear detections at each rub object, divided by the number of sample occasions each rub object was visited. We used AIC to identify the most parsimonious model for each sex among 11 candidate models (Supporting Information Table S1). We calculated RSFs only within the area of inference (Supporting Information Figure S1).

Third, we extrapolated male (48.3 bears/1,000 km^2^, 95% CI: 40.2–57.3) and female (72.3/1,000 km^2^, 95% CI: 60.3–87.5) density estimates calculated for GNP (Stetz et al., 2014) to our reference area. We related abundance to habitat quality (i.e., RSF score) following Boyce and McDonald (1999). We completed this separately for males and females. We assumed the reference area was at carrying capacity because of a low human footprint, hunting restrictions, and proximity to GNP (i.e., source area; Stetz et al., 2014). These factors contribute to the long‐term equilibrium abundance (population carrying capacity *K* where d*N*/d*t* = 0) of a site. Because Beauvais Provincial Park does not share a boundary with GNP, is small (11.6 km^2^) and is separated from WLNP by 25 km, we did not include Beauvais in the reference area. To extrapolate to an area with lower equilibrium abundance, we assume the same factors influencing variation in *K* within WLNP would influence equilibrium abundance at sites at lower population sizes outside WLNP. Using the top RSF model, we reclassified scaled RSF scores into 10 bins. Because selection is proportional to the probability of use, we calculated the relative probability of use as:

$$U\left(x_{i}\right)=\frac{w\left(x_{i}\right)A\left(x_{i}\right)}{\sum_{j}w\left(x_{j}\right)A\left(x_{j}\right)},$$

where *w*(*x*_*i*_) is the midpoint probability for an RSF bin and *A*(*x*_*i*_) is the area for a vector of *i* habitat variables. For the *i*th habitat class, we calculated the expected number of bears as ${\hat{N}}_{i}=\hat{N}\times U\left(x_{i}\right)$ where $\hat{N}$ is the estimated population size for WLNP, and density is ${\hat{D}}_{i}={\hat{N}}_{i}/A_{i}\left(x_{i}\right)$.

Fourth, based on density and habitat associations in WLNP, we extrapolated *D* across our area of inference. We approximated confidence intervals (CI) by extrapolating based on the 95% CI of the abundance estimate for GNP. We used *k*‐fold cross‐validation to measure the predictive ability of the RSF (Boyce, Vernier, Nielsen, & Schmiegelow, 2002). We partitioned the data into 10 folds and tested the association between the frequency of presence observations in 10 RSF bin ranks. We did this 10 times and used the averaged Spearman's rank correlation coefficient (${\overset{¯}{r}}_{s}$) to evaluate the predictive success of each RSF model (Boyce et al., 2003).

## 4 RESULTS

### 4.1 Hair collection

In 2013, we submitted 4,554 hair samples to WGI for analysis, resulting in 306 detections of 126 males and 177 detections of 101 female black bears (Table 1). We detected black bears at 52% of the rub objects (*n *=* *466). In 2014, we submitted 3,912 hair samples, resulting in 294 detections of 122 males and 168 detections of 100 females (Table 1). We detected black bears at 48% of the rub objects (*n *=* *444). Across both years, we identified 347 black bears (186 males, 161 females), 107 of which were detected in both years. See the Supporting Information for detailed genetic results.

### 4.2 SECR models

**Figure 2:**
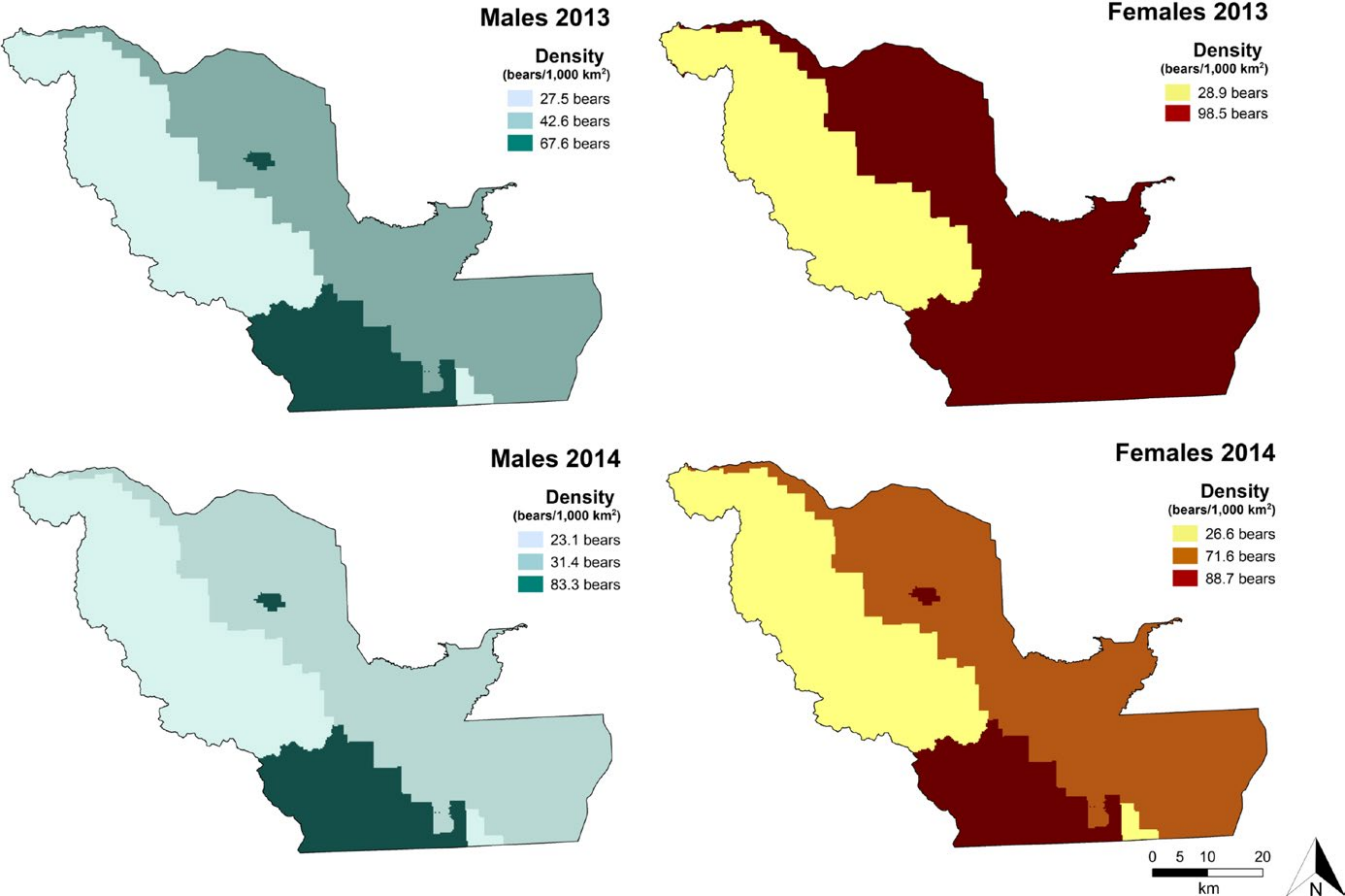
Surface densities derived from top‐performing male and female black bear spatially explicit capture–recapture models in southwestern Alberta (2013–2014)

The top detection model from step 1 for males in 2013 and 2014 included *λ*_0_ covariates traptype, bk, and *T* (Table 3). The *σ* covariate included *T*. The top density models for males from step 2 in 2013 and 2014 allowed density to vary by land tenure, with density lowest on Crown lands and highest on protected lands (Table 3). Relative standard error (RSE) of the density estimate was 8.7% for males in 2013 and 8.5% in 2014. Male beta coefficients for Crown lands were negative relative to protected lands, our reference level (2013: *β*_private_ = −0.45, *SE* = 0.27; *β*_Crown_ = −0.89, *SE* = 0.28; 2014: *β*_private_ = −1.0, *SE* = 0.26; *β*_Crown_ = −1.3, *SE* = 0.27; Figure 2). Sigma (*σ*) increased over time (2013: *β*_*T*_ = 0.11, *SE* = 0.03; 2014: *β*_*T*_ = 0.03, *SE* = 0.03; Table 4). Detection varied by trap type (Table 4).

**Table 3:** Model selection for spatially explicit capture–recapture models using detection data for black bears in southwestern Alberta

Sex	Year	Model Step	Model description	*K*	LL	AICc	∆AICc	*w*_i_
Males	2013	1	*D *~ 1 *λ*_0_ * *~ traptype + bk + *T σ *~ *T*	8	−1,750.67	3,518.58	0.00	1.00
		2	*D *~ tenure *λ*_0_ * *~ traptype + bk + *T σ *~ *T*	10	−1,745.77	3,513.46	0.00	0.60
			*D *~ harvest *λ*_0_ * *~ traptype + bk + *T σ *~ *T*	9	−1,748.21	3,515.97	2.52	0.17
			*D *~ tertiary rd *λ*_0_ * *~ traptype + bk + *T σ *~ *T*	9	−1,748.39	3,516.32	2.87	0.14
			*D *~ burn *λ*_0_ * *~ traptype + bk + *T σ *~ *T*	9	−1,750.08	3,519.71	6.25	0.03
			*D *~ ndvi *λ*_0_ * *~ traptype + bk + *T σ *~ *T*	9	−1,750.35	3,520.25	6.82	0.02
			*D *~ water *λ*_0_ * *~ traptype + bk + *T σ *~ *T*	9	−1,750.54	3,520.63	7.17	0.02
			*D *~ rddens *λ*_0_ * *~ traptype + bk + *T σ *~ *T*	9	−1,750.66	3,520.88	7.42	0.01
			*D *~ can_cov *λ*_0_ * *~ traptype + bk + *T σ *~ *T*	9	−1,750.83	3,521.21	7.76	0.01
Males	2014	1	*D *~ 1 *λ*_0_ * *~ traptype + bk + *T σ *~ *T*	8	−1,710.17	3,437.61	0.00	0.81
			*D *~ 1 *λ*_0_ * *~ *T σ *~ 1	4	−1,716.95	3,442.24	4.63	0.08
			*D *~ 1 *λ*_0_ * *~ GB + *T σ *~ 1	5	−1,716.60	3,443.71	6.11	0.04
			*D *~ 1 *λ*_0_ * *~ *T σ *~ *T*	5	−1,716.66	3,443.84	6.24	0.04
			*D *~ 1 *λ*_0_ * *~ *T* + habitat + bk *σ *~ 1	11	−1,710.26	3,444.92	7.31	0.02
			*D *~ 1 *λ*_0_ * *~ GB + *T* + traptype *σ *~ 1	7	−1,715.72	3,446.42	8.82	0.01
		2	*D *~ tenure *λ*_0_ * *~ traptype + bk + *T σ *~ *T*	10	−1,698.54	3,419.05	0.00	0.81
			*D *~ tertiary rd *λ*_0_ * *~ traptype + bk + *T σ *~ *T*	9	−1,701.15	3,421.90	2.85	0.19
Females	2013	1	*D *~ 1 *λ*_0_ * *~ traptype + bk *σ *~ traptype	8	−950.88	1,919.33	0.00	1.00
		2	*D *~ harvest *λ*_0_ * *~ traptype + bk *σ *~ traptype	9	−940.50	1,900.97	0.00	0.61
			*D *~ tenure *λ*_0_ * *~ traptype + bk *σ *~ traptype	10	−939.72	1,901.89	0.92	0.39
Females	2014	1	*D *~ 1 *λ*_0_ * *~ traptype + bk *σ *~ traptype	8	−950.88	1,919.33	0.00	1.00
		2	*D *~ tenure *λ*_0_ * *~ traptype + bk *σ *~ traptype	10	−956.16	1,934.79	0.00	0.99
			*D *~ tertiary rd *λ*_0_ * *~ traptype + bk *σ *~ traptype	9	−961.90	1,943.79	9.00	0.01

AICc: Akaike information criterion corrected for small sample sizes; GB: grizzly bear; *K*: number of model parameters; LL: log‐likelihood.

In step 1, we identified the top *λ*_0_ and *σ* covariates. In step 2, we used the step 1 model as the base model on which to build heterogeneous density models. Models that did not receive any model weight (*w*_*i*_ = 0) are not shown here. See Section 2 for variable definitions.

**Table 4:** Parameter estimates from top SECR models for male and female black bears in southwestern Alberta in 2013 and 2014

Year	Sex	Covariate	Levels	Density	95% CI	*λ*_0_ (*SE*)	*σ* (*SE*)
2013	Male	Tenure	Park	67.6	46.5–95.6	Traptype_rub_ bk_0_: 0.029 (0.004)	*T* = 0: 3.8 (0.22)
			Private	42.6	30.9–58.7	Traptype_rub_ bk_1_: 0.160 (0.043)	*T* = 7: 8.3 (1.34)
			Crown	27.5	19.0–39.8	Traptype_opp_ bk_0_: 0.032 (0.078)	
						Traptype_opp_ bk_1_: 0.178 (0.451)	
						Traptype_fence_ bk_0_: 0.013 (0.006)	
						Traptype_fence_ bk_1_: 0.073 (0.036)	
	Female	Harvest Density	Max	28.9	16.1–51.9	Traptype_rub_ bk_0_: 0.018 (0.003)	Traptype_rub_: 1.94 (0.15)
			Min	98.5	73.8–131.4	Traptype_rub_ bk_1_: 0.080 (0.020)	Traptype_fence_: 8.48 (3.31)
			Mean	88.8	67.5–116.8	Traptype_opp_ bk_0_: 9E‐05 (8E‐05)	Traptype_opp_: 95.0 (763.5)
						Traptype_opp_ bk_1_: 0.0004 (0.0004)	
						Traptype_fence_ bk_0_: 0.0004 (0.0004)	
						Traptype_fence_ bk_1_: 0.002 (0.002)	
2014	Male	Tenure	Park	83.3	61.5–112.7	Traptype_rub_ bk_0_: 0.020 (0.003)	*T* = 0: 4.62 (3.08)
			Private	31.4	21.9–44.9	Traptype_rub_ bk_1_: 0.067 (0.023)	*T* = 7: 5.63 (1.02)
			Crown	23.1	15.3–34.9	Traptype_opp_ bk_0_: 0.012 (0.010)	
						Traptype_opp_ bk_1_: 0.040 (0.039)	
						Traptype_fence_ bk_0_: 0.027 (0.007)	
						Traptype_fence_ bk_1_: 0.001 (0.0001)	
	Female	Tenure	Park	88.7	57.4–137.0	Traptype_rub_ bk_0_: 0.005 (0.001)	Traptype_rub_: 3.74 (0.31)
			Private	71.6	48.9–104.8	Traptype_rub_ bk_1_: 0.041 (0.013)	Traptype_fence_: 1.38 (0.39)
			Crown	26.6	15.4–45.9	Traptype_opp_ bk_0_: 0.0001 (9.2E‐05)	Traptype_opp_: 113.73 (1,351.37)
						Traptype_opp_ bk_1_: 0.001 (0.001)	
						Traptype_fence_ bk_0_: 0.027 (0.014)	
						Traptype_fence_ bk_1_: 0.225 (0.101)	

bk: previous capture of *x* individual (0,1); Max: maximum harvest density; Mean: mean harvest density; Min: minimum harvest density; SECR: spatially explicit capture–recapture; *T*: linear time trend.

Densities are reported in bears/1,000 km^2^, *λ*_0_ is the cumulative hazard of detection, and *σ* is the spatial scale parameter (km).

The top detection models from step 1 for females in 2013 and 2014 included *λ*_0_ covariates trap type and bk, and *σ* covariate trap type (Table 4). The top density model for females from step 2 in 2013 included harvest. There was a negative relationship between harvest and black bear density for females (*β*_hunt_ = −0.27, *SE* = 0.07; Figure 2, Table 4). RSE of the density estimate was 13.4% for females in 2013 and 13.5% in 2014. The top density model from step 2 for females in 2014 included land tenure (Table 4). Results indicated an inverse relation between Crown lands and female black bear density in 2014 (*β*_Crown_ = −1.20, *SE* = 0.34; *β*_private_ = −0.21, *SE* = 0.28; Figure 2).

For each sex and year, we predicted the density surface at each mask point and used discrete summation to calculate abundance within each land tenure. Abundance estimates indicate female‐biased sex ratios on private land (2.3F:1M; Table 5).

**Table 5:** Male and female abundance estimates from SECR models in southwestern Alberta

Sex	Year	Tenure	Abundance	95% CI	Sex ratio (F/M)
Males	2013	Park	33.4	23.3–47.7	
		Private	79.6	58.1–109.2	
		Crown	31.8	23.4–48.8	
	2014	Park	41.7	30.8–56.3	
		Private	59.1	41.6–83.7	
		Crown	28.5	19.0–42.7	
Females	2013	Park	49.8	37.4–66.4	1.5
		Private	183.0	137.3–243.8	2.3
		Crown	37.9	22.2–64.5	1.2
	2014	Park	44.4	28.9–68.2	1.1
		Private	133.6	91.8–194.3	2.3
		Crown	32.7	19.2–55.8	1.1

Note

SECR: spatially explicit capture–recapture.

### 4.3 Resource‐selection functions

The global RSF indicated rub objects were in areas with high NDVI values, low to mid‐elevations, and not in agricultural areas such as cropland and year‐round cattle pastures. When the lowest three RSF bins were excluded, the area of inference was reduced to 2,364 km^2^ (Supporting Information Figure S1). We removed the lowest three bins because there was a large break between the third and fourth lowest bin, which we interpreted as a natural cutoff for defining the area of inference.

**Figure 3:**
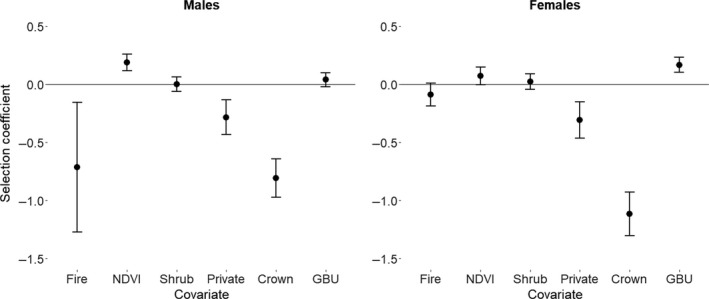
Scaled beta coefficients for top resource‐selection function models for male and female black bears in southwestern Alberta, Canada. We compared detection locations, and associated habitat covariates, to the full set of rub objects in 2013 and 2014. Error bars represent standard error. GBU: grizzly bear use; NDVI: Normalized Difference Vegetation Index

**Figure 4:**
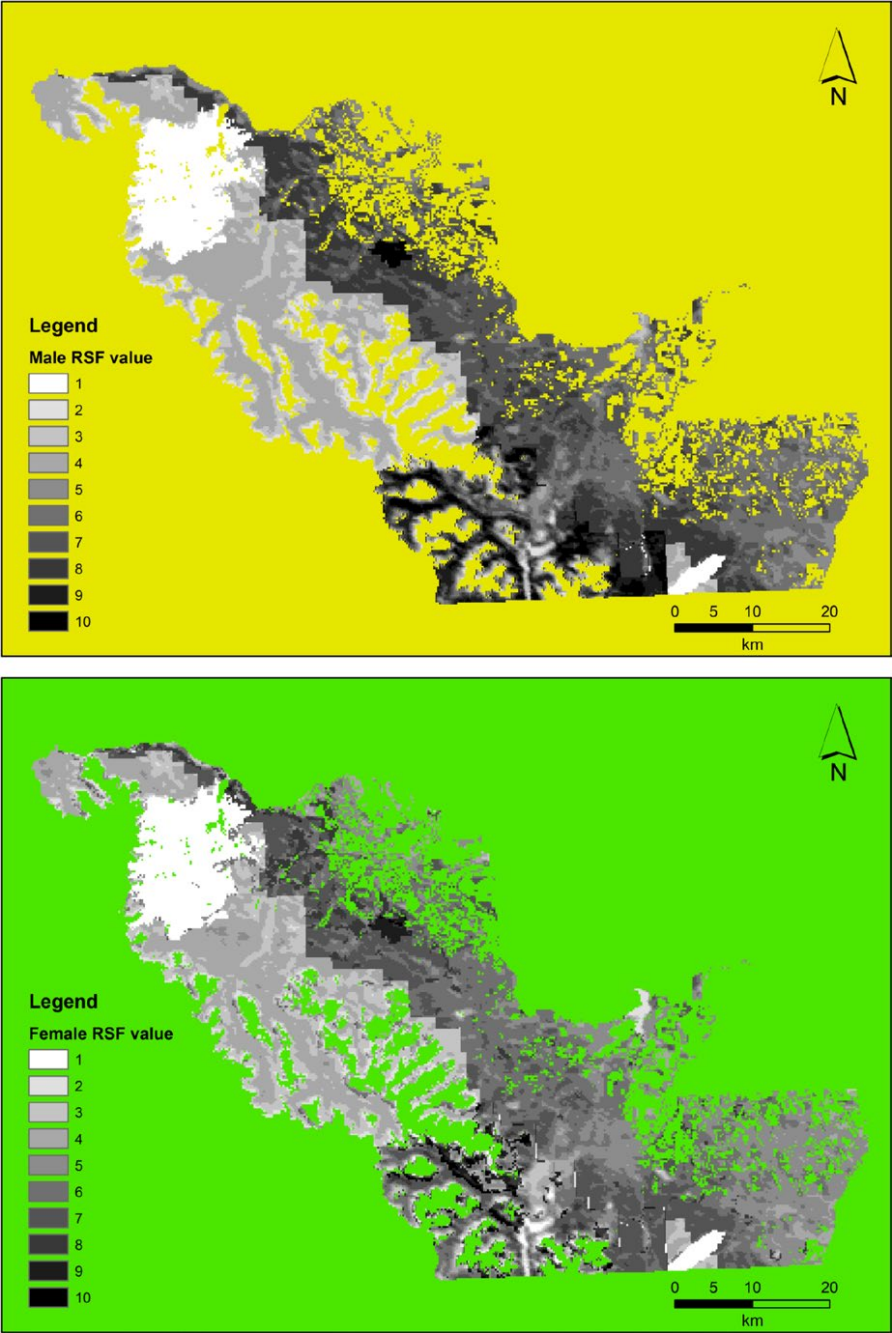
Spatial variation in top‐performing male (top) and female (bottom) black bear resource‐selection function (RSF) models in southwestern Alberta (2013–2014). Top models for males and females included spatial covariates burned areas <20 years old, Normalized Difference Vegetation Index, shrub cover, land tenure, and grizzly bear use

Across our study area, we detected male black bears at 407 locations in 2013 and 2014. Male black bears selected higher NDVI, avoided burned areas, and avoided Crown and private land relative to protected lands (Figures 3 and 4, ${\overset{¯}{r}}_{s}$ = 0.70). Other covariates in the top model had no apparent effect (CI overlapped zero). We had 323 locations for female black bears in 2013 and 2014. Female black bears selected higher canopy cover and GBU and avoided private and Crown lands (Figures 3 and 4, ${\overset{¯}{r}}_{s}$ = 0.74). Other covariates in the top model had no apparent effect.

**Figure 5:**
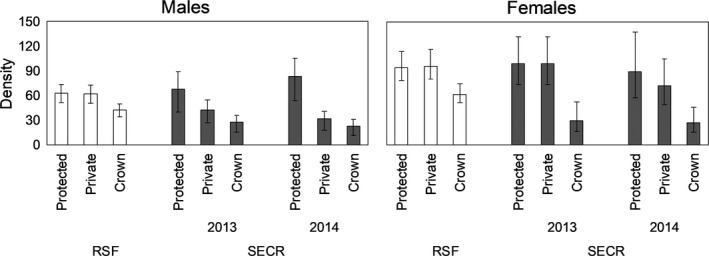
Spatially explicit capture–recapture (SECR) and resource‐selection function (RSF)‐derived densities for number of male and female black bears in the southwestern Alberta in 2013 and 2014. For RSF densities, reference area densities were extrapolated from Glacier National Park (Stetz et al., 2014). Error bars represent 95% CI. Density is reported in bears/1,000 km^2^

We used an independent GNP abundance estimate (Stetz et al., 2014) for the reference area. Extrapolating density based on RSF values, black bear density was 62.0 males/1,000 km^2^ (95% CI: 51.6–73.7) on private lands and 42.7 males/1,000 km^2^ (95% CI: 35.5–73.7) on Crown lands (Figure 5). Density in the protected area was 62.8 males/1,000 km^2^ (95% CI: 52.3–74.6). Female black bear density was 95.7 females/1,000 km^2^ (95% CI: 79.8–115.9) on private lands and 61.4 females/1,000 km^2^ (95% CI: 51.2–74.4) on Crown lands (Figure 5). Density in the protected area was 94.1 females/1,000 km^2^ (95% CI: 78.4–113.8). RSF values were highly structured by land tenure with Crown lands having the lowest frequency and protected lands having the highest frequency of high‐quality habitat (Supporting Information Figure S2).

## 5 DISCUSSION

We generated the first density and abundance estimates for black bears in southwestern Alberta. From SECR models, land tenure best explained spatial variation in male and female black bear density, except females in 2013 where harvest was the best predictor covariate for density. Land tenure was also an important predictor covariate for RSF models. A large‐scale covariate like land tenure can be confounded because it may be describing more than just “land tenure.” For example, land tenure encompasses several habitat differences such as road density, with protected lands having the lowest road density and private land having the highest road density. However, support for this covariate might be related to the multiplicative effect of road density and harvest intensity, which could explain why the individual covariates “roads” and “harvest density” did not perform better than land tenure (except for 2013 females).

For RSF‐derived estimates, male and female density was highest in the reference area, which was consistent with results from SECR modeling. As well, female densities were higher than male densities, meeting our original predictions. On average, male densities in the protected lands were 3.1 times and 2.1 times higher than Crown and private lands, respectively; female densities in the protected lands were 3.4 and 2.3 times higher than Crown and private lands, respectively. While some protected areas in North America, particularly mountain parks, do not contain high‐quality habitats (Jenkins et al., 2015; Joppa & Pfaff, 2009), our results suggest that protected lands in our study area contain high‐quality habitats because RSF scores and relative densities were highest there. Both RSF and SECR models point to land tenure as the most important predictor of black bear density, followed by harvest density for SECR models, and habitat productivity and recently burned areas for RSF models. Both males and females showed avoidance of recently burned areas, though female avoidance was not significant. Wildfires can produce high‐quantity and high‐quality bear foods; primary bear foods such as buffalo berry (*Shepherdia canadensis*), Saskatoon berry (*Amelanchier alnifolia*), and species of *Vaccinium* can produce up to 20 times more fruit when comparing adjacent burned and unburned mature forests (Hamer, 1996; Young & Beecham, 1986). Recovery from wildfire depends on site productivity, precipitation levels following the burn, forest type, and intensity of the wildfire, among other factors. Fire can have prolonged negative effects on forest cover, for example, which is important escape terrain for black bears. In turn, black bears have been shown to avoid large, high‐intensity burned areas with little protective cover from sympatric grizzly bears, which select for relatively treeless burned areas (McLellan, 2011). Even in areas without grizzly bears, wildfires can have demographic consequences on black bears beyond direct mortalities (Singer, Schreier, Oppenheim, & Garton, 1989), such as reduced cub survival and sex ratios skewed toward males (Cunningham & Ballard, 2004). In our study area, the wildfire that we suspect is driving our top RSF models was a 177‐km^2^ high‐intensity fire that burned on Crown lands in 2003. The Lost Creek Fire continues to have little protective tree cover for black bears, and with high grizzly bear densities for interior populations (Morehouse & Boyce, 2016), this may be a contributing factor to reduced black bear densities on Crown lands.

Sex ratios were strongly female‐biased on private lands, while not so on Crown and protected lands (Table 5). We did not predict that there would be sex‐specific spatial structuring in our study. Black bears, and large mammals in general, often exhibit a female bias in un‐hunted populations (Clutton‐Brock & Iason, 1986), and female black bears drive population growth because one male can impregnate many females (Beston, 2011). Hunting can exacerbate this bias, particularly for bears, where males are disproportionately harvested because of hunter selection for increased body size, legal protection for females with cubs, and larger home‐range size for males (Bunnell & Tait, 1980; Garshelis, 1990; Miller, 1990). In our study area, we speculate that skewed sex ratios are a result of females emigrating out of protected lands that have a high density of male black bears, and to an area with lower female harvest relative to Crown lands.

**Figure 6:**
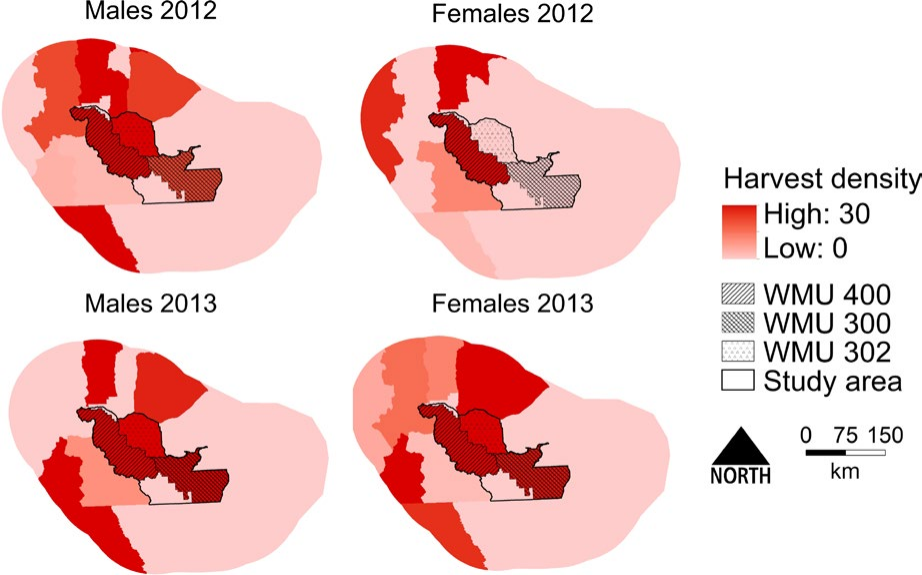
Harvest density (individuals/1,000 km^2^) for male and female black bears the year prior to non‐invasive genetic sampling in southwestern Alberta (2013–2014). Wildlife management unit (WMU) 400 is on Crown land and WMU 303 and 302 are on private land

Females will select habitats to minimize predation risk to their offspring (Ruckstuhl & Neuhaus, 2002) or can be excluded from high‐quality habitats by males (Craighead, Sumner, & Mitchel, 1995). Because protected lands have the highest male black bear densities of all land tenures, females might be dispersing to areas with a lower density of males where competition for high‐quality resources is diminished. In our study area, black bears can be harvested on both Crown and private lands, but we saw higher female harvest on Crown lands (Figure 6). In Alberta, reporting of black bear harvests is not required and we assume harvest rates reported by the provincial government underestimate actual harvest rates. However, we suspect higher harvest on Crown lands are driven by a variety of factors. Southwestern Alberta is a mosaic of landowners, and hunters require individual landowner permission to hunt on private lands. In contrast, permission is not required to hunt on Crown lands and hunters only need to purchase a hunting license. Local biologists and Fish and Wildlife Officers also indicate that many black bears in the fall are killed opportunistically on Crown lands when hunters are looking for other open‐season animals. Overall road densities are higher on private lands relative to Crown lands. However, most hunt areas in our study area are accessed via low‐traffic tertiary roads. On Crown lands, tertiary road densities are higher (0.31 km/km^2^) relative to private lands (0.24 km/km^2^), increasing access opportunities for hunters. Thus, it is not only logistically easier to hunt on Crown lands, and hunters may opportunistically hunt black bears, there is also increased road access to hunting areas on Crown lands.

In our study, we found differences in sex ratios between land tenures and this could have long‐term consequences for black bears. Because Crown lands have higher harvest rates for black bears, and if there are low recruitment rates, we could expect that black bears on Crown lands would contribute less to population growth (Novaro, Funes, & Walker, 2005). In Alberta, Crown lands are often considered “core” black bear habitats (Webb, Morcos, Allen, & Frame, 2016) and wildlife managers trust these areas to be population sources, rather than population sinks, albeit without empirical data on population trend, abundance, or density. Cautious interpretation is required of our results, however, because without knowing population age structure, differences or changes in sex ratios could be misinterpreted as a population decline, for example, when the population is dominated by a specific age cohort (e.g., subadults; Garshelis, 1990). Despite this caveat, our results indicate spatial structuring of mortality and we suggest further monitoring to assess demographic consequences of high harvest. Improvement to provincial harvest reporting (e.g., required reporting) would help to gain insight, particularly for non‐licensed hunting on private land.

Animal densities are usually related to habitat selection (Boyce et al., 2016), and RSFs can be used to describe this relationship (Johnson et al., 2006; Manly et al., 2002). Our SECR and RSF‐derived abundance produced density estimates with generally overlapping confidence intervals (with exceptions). We draw some common conclusions from the concurrent modeling. First, RSF‐derived density estimates had smaller 95% CIs than the SECR density estimates. This likely stems from the high variance of ratio estimators (Czaplewski et al., 1983) such as capture–recapture estimators, and the additional parameterizations of *σ* and *λ*_0_. RSFs do not account for imperfect detection; they only compare used locations to locations where an animal could have been. In contrast, SECR accounts for animals we did not detect by estimating un‐observed bear home‐range centers. The accuracy and precision of SECR abundance and density estimates depend on the ability to model factors influencing *σ* and *λ*_0_ (Whittington & Sawaya, 2015). Thus, it is reasonable that with low cumulative hazard of detection, SECR models would generate larger variance estimates than the RSF models.

Second, RSF and SECR density estimates showed similar trends in density by land tenure with density highest on protected lands and lowest on Crown lands. However, RSF models estimated higher black bear densities on Crown lands relative to SECR models. SECR and RSF male black bear densities had overlapping confidence intervals on protected lands in both years, and private and Crown lands only in 2013. SECR and RSF female black bear densities had overlapping confidence intervals on protected and private lands in both years, and Crown lands only in 2013 (Figure 5). There are possibly several reasons for inconsistencies between years and land tenure types. Black bears are generalists and could have a looser relationship between habitat quality and selection than habitat specialists, making the RSF‐abundance extrapolation more subject to errors in identifying selected habitats. In particular, the proportion of black bears detections on Crown lands was lowest (M: 28%; F: 22%) relative to protected (M: 33%; F: 37%) and private lands (M: 38%; F: 41%). This relative data deficiency could have exacerbated the misidentification of black bear habitat, leading to unexpectedly high RSF‐derived densities on Crown lands.

Alternatively, *λ*_0_ values from our SECR models were higher in 2014 relative to 2013 for males and females. We suggest that factors influencing a reduced cumulative hazard of detection (or capture probabilities) also could influence the accuracy of an RSF. For example, if 2014 was a food‐poor year and black bears were moving longer distances to search for alternative food sources (explaining the increase in *σ* and reduction in *λ*_0_), the strength of the selection–habitat quality relationship could be diminished (Nielsen, McDermid, Stenhouse, & Boyce, 2010). This could explain why RSF and SECR confidence intervals did not overlap in 2014.

However, perhaps the most obvious reason for differences between RSF and SECR estimates is that we used abundance estimates from GNP as our reference area; thus, our RSF‐derived estimates were dependent on GNP estimates. Density in GNP could have changed substantially between 2004 and 2013–2014, when our data collection occurred. Over those 9–10 years, extrinsic (e.g., wildfire, precipitation) or intrinsic (e.g., age structure, sex bias) factors could have caused black bear populations to increase. For this reason, we recommend managers use SECR‐derived density and abundance estimates when feasible. However, in line with objective 2, we wanted to identify spatial covariates driving black bear density. Using two parallel methods allowed us to explore differential drivers of density.

While we advocate for the use of SECR models for density estimates, it is not our intent to undermine the utility of RSF models. Many wildlife agencies focus on creating habitat‐based models, such as RSFs, which are useful in identifying high‐quality or critical habitats. For example, a variation on the RSF‐abundance extrapolation was used in British Columbia to estimate grizzly bear abundance (Fuhr & Demarchi, 1990). In our study, spatial variation in density between RSF and SECR methods were consistent, generally protected lands had highest density and Crown lands had lowest density. Our results reinforce the importance of habitat and land use in estimating population size, even for a generalist species such as the black bear. Further, the reduced laboratory costs for only identifying males and females used in the RSF (vs. identifying individuals for SECR) could be attractive to agencies and institutions with restricted budgets. We acknowledge that the RSF‐based method requires an independent abundance estimate for the reference area, and depending on the species, time, distribution, and habitat type(s), these estimates might not exist or be suitable for extrapolation.

Black bear monitoring studies are often spatially and temporally isolated (Beston, 2011). With recent abundance and density estimates for GNP (Stetz et al., 2014), our study adds demographic information to a shared population of black bears but on a multi‐use landscape. The only previous estimate for black bear abundance in southwestern Alberta (Gunson & Markham, 1993) was derived without variance estimates, which precludes comparison and inferences about population changes (Miller, 1990). As a coarse‐level comparison, our estimates were in the range of reported interior black bear densities where sympatric with grizzly bears (mean = 164 bears/1,000 km^2^), although the range of densities is high (range = 9–450/1,000 km^2^; Mattson, Herrero, & Merrill, 2005). While our black bears density estimates have low relative bias (RSE ranged from 8.5% to 13.5%), precision could be improved with a secondary data source (e.g., hair traps; Boulanger et al., 2008), particularly for females where RSEs were higher than for males. This likely stems from a lower cumulative hazard of detection for females relative to males.

If private land in our study area is acting as a spatial refuge for female black bears, our results suggest the need for active management on Crown lands where black bear densities are lowest. In particular, harvest and management differences by land tenure, such as road densities, should be targeted. For example, grizzly bear densities were higher in British Columbia where motorized vehicle access was restricted (Proctor et al., 2018).

Black bear rubbing on a tree in southwestern Alberta, Canada. Photo credit: Ryan Peruniak

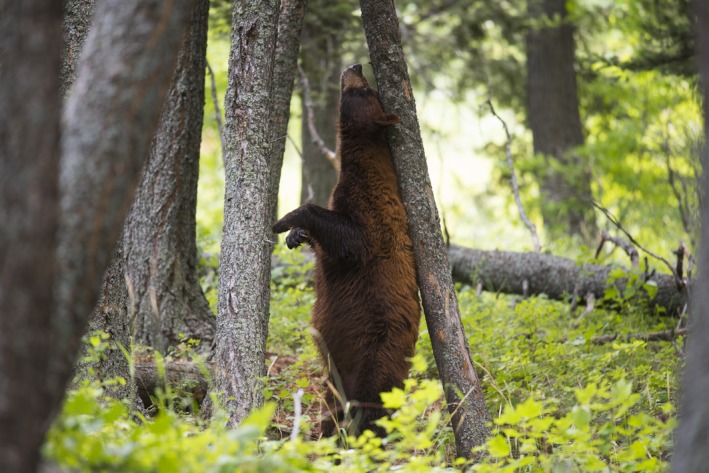

## CONFLICT OF INTEREST

None declared.

## AUTHORS’ CONTRIBUTIONS

AEL and ATM conceived the ideas and designed methods; AEL and ATM collected the data; AEL analyzed the data; AEL wrote the manuscript; MSB supervised research and analysis.

## DATA ACCESSIBILITY

RSF and capture files can be found at the University of Alberta Dataverse website doi.org/10.7939/DVN/YT7ZZW

## References

[ece34617-bib-0001] Apps, C. D. , McLellan, B. N. , & Woods, J. G. (2006). Landscape partitioning and spatial inferences of competition between black and grizzly bears. Ecography, 29, 561–572. 10.1111/j.0906-7590.2006.04564.x

[ece34617-bib-0002] Benítez‐López, A. , Alkemade, R. , & Verweij, P. A. (2010). The impacts of roads and other infrastructure on mammal and bird populations: A meta‐analysis. Biological Conservation, 143, 1307–1316. 10.1016/j.biocon.2010.02.009

[ece34617-bib-0003] Berger, J. (2004). The last mile: How to sustain long distance migration in mammals. Conservation Biology, 18, 320–331. 10.1111/j.1523-1739.2004.00548.x

[ece34617-bib-0004] Beston, J. (2011). Variation in life history and demography of the American black bear. Journal of Wildlife Management, 75, 1588–1596. 10.1002/jwmg.195

[ece34617-bib-0006] Boulanger, J. , Kendall, K. C. , Stetz, J. B. , Roon, D. A. , Waits, L. P. , & Paetkau, D. (2008). Multiple data sources improve DNA‐based mark‐recapture population estimates of grizzly bears. Ecological Applications, 18, 577–589. 10.1890/06-1941.1 18488618

[ece34617-bib-0007] Boyce, M. S. , Johnson, C. J. , Merrill, E. H. , Nielsen, S. E. , Solberg, E. J. , & van Moorter, B. (2016). Can habitat selection predict abundance? Journal of Animal Ecology, 85, 11–20. 10.1111/1365-2656.12359 25786026

[ece34617-bib-0008] Boyce, M. S. , Mao, J. S. , Merrill, E. H. , Fortin, D. , Turner, M. G. , Fryxell, J. , & Turchin, P. (2003). Scale and heterogeneity in habitat selection by elk in Yellowstone National Park. Ecoscience, 10, 421–431. 10.1080/11956860.2003.11682790

[ece34617-bib-0009] Boyce, M. , & McDonald, L. (1999). Relating populations to habitats using resource selection functions. Trends in Ecology and Evolution, 14, 268–272. 10.1016/S0169-5347(99)01593-1 10370262

[ece34617-bib-0010] Boyce, M. S. , Vernier, P. R. , Nielsen, S. E. , & Schmiegelow, F. K. A. (2002). Evaluating resource selection functions. Ecological Modelling, 157, 281–300. 10.1016/S0304-3800(02)00200-4

[ece34617-bib-0011] Boyce, M. S. , & Waller, J. S. (2003). Grizzly bears for the Bitterroot: Predicting potential abundance and distribution. Wildlife Society Bulletin, 31, 670–683.

[ece34617-bib-0012] Bunnell, F. L. , & Tait, D. E. N. (1980). Bears in models and in reality: Implications to management. Bears: Their Biology and Management, 4, 15–23.

[ece34617-bib-0013] Burnham, K. , & Anderson, D. (2002). Model selection and multimodal inference: A practical information‐theoretic approach (488 pp.). New York, NY: Springer 10.1007/b97636

[ece34617-bib-0014] Choquet, R. , Lebreton, J. D. , Gimenez, O. , Reboulet, A. M. , & Pradel, R. (2009). U‐CARE: Utilities for performing goodness of fit tests and manipulating CApture‐REcapture data. Ecography, 32, 1071–1074. 10.1111/j.1600-0587.2009.05968.x

[ece34617-bib-0015] Clapham, M. , Nevin, O. T. , Ramsey, A. D. , & Rosell, F. (2012). A hypothetico‐deductive approach to assessing the social function of chemical signalling in a non‐territorial solitary carnivore. PLoS One, 7, e35404 10.1371/journal.pone.0035404 22530018 PMC3329431

[ece34617-bib-0016] Clutton‐Brock, T. H. , & Iason, G. R. (1986). Sex ratio variation in mammals. Quarterly Review of Biology, 61, 339–374. 10.1086/415033 3532167

[ece34617-bib-0017] Craighead, J. J. , Sumner, J. S. , & Mitchel, J. A. (1995). The grizzly bears of Yellowstone: Their ecology in the Yellowstone Ecosystem, 1959–1992 (556 pp.). Washington, DC: Island Press.

[ece34617-bib-0018] Cunningham, S. C. , & Ballard, W. B. (2004). Effects of wildfire on black bear demographics in central Arizona. Wildlife Society Bulletin, 32, 928–937. 10.2193/0091-7648(2004)032[0928:EOWOBB]2.0.CO;2

[ece34617-bib-0019] Czaplewski, R. L. , Crowe, D. M. , & McDonald, L. L. (1983). Sample sizes and confidence intervals for wildlife population ratios. Wildlife Society Bulletin, 11, 121–128.

[ece34617-bib-0020] Efford, M. (2004). Density estimation in live‐trapping studies. Oikos, 106, 598–610. 10.1111/j.0030-1299.2004.13043.x

[ece34617-bib-0021] Efford, M. (2016). secr: Spatially explicit capture‐recapture models. R package version 2.10.2. Retrieved from https://cran.r-project.org/package=secr

[ece34617-bib-0022] Efford, M. G. , Borchers, D. L. , & Byrom, A. E. (2009). Density estimation by spatially explicit capture‐recapture: Likelihood‐based methods In Thomson D. L., Cooch E. G., & Conroy M. J. (Eds.), Modeling demographic processes in marked populations (p. 255–269). New York, NY: Springer Science and Business Media 10.1007/978-0-387-78151-8

[ece34617-bib-0023] Efford, M. G. , & Fewster, R. M. (2013). Estimating population size by spatially explicit capture‐recapture. Oikos, 122, 918–928. 10.1111/j.1600-0706.2012.20440.x

[ece34617-bib-0024] Efford, M. G. , & Mowat, G. (2014). Compensatory heterogeneity in spatially explicit capture–recapture data. Ecology, 95, 1341–1348. 10.1890/13-1497.1 25000765

[ece34617-bib-0025] Ennis, S. , & Gallagher, T. F. (1994). A PCR‐based sex‐determination assay in cattle based on the bovine amelogenin locus. Animal Genetics, 25, 425–427. 10.1111/j.1365-2052.1994.tb00533.x 7695123

[ece34617-bib-0026] Fletcher, D. , Lebreton, J. D. , Marescot, L. , Schaub, M. , Gimenez, O. , Dawson, S. , & Slooten, E. (2012). Bias in estimation of adult survival and asymptotic population growth rate caused by undetected capture heterogeneity. Methods in Ecology and Evolution, 3, 206–216. 10.1111/j.2041-210X.2011.00137.x

[ece34617-bib-0027] Fuhr, B. , & Demarchi, D. A. (1990). A methodology for grizzly bear habitat assessment in British Columbia. Wildlife Bulletin, ISSN 0829‐9560; no. B‐67. Victoria, BC: British Columbia Ministry of Environment.

[ece34617-bib-0028] Garshelis, D. L. (1990). Monitoring effects of harvest on black bear populations in North America: A review and evaluation of techniques. Eastern Workshop of Black Bear Research and Management, 10, 102–144.

[ece34617-bib-0029] Garshelis, D. L. , & Hristienko, H. (2006). State and provincial estimates of American black bear numbers versus assessments of population trend. Ursus, 17, 1–7. 10.2192/1537-6176(2006)17[1:SAPEOA]2.0.CO;2

[ece34617-bib-0030] Gunson, J. R. & Markham, B.J. (1993). Management plan for black bears in Alberta. ISNB: 0-86499-945-3, Edmonton, AB: Alberta Environmental Protection Fish and Wildlife Services.

[ece34617-bib-0031] Hamer, D. (1996). Buffaloberry [*Shepherdia canadensis* (L.) Nutt.] fruit production in fire‐successional bear feeding sites. Journal of Range Management, 49, 520–529. 10.2307/4002293

[ece34617-bib-0032] Herrero, S. (1978). A comparison of some features of the evolution, ecology, and behavior of black and grizzly/brown bears. Carnivora, 1, 7–16.

[ece34617-bib-0033] Holm, G. , Lindzey, F. , & Moody, D. (1999). Interactions of sympatric black and grizzly bears in northwest Wyoming. Ursus, 11, 99–108.

[ece34617-bib-0034] Jenkins, C. N. , Van Houtan, K. S. , Pimm, S. L. , & Sexton, J. O. (2015). US protected lands mismatch biodiversity priorities. Proceedings of the National Academy of Sciences USA, 112, 5081–5086. 10.1073/pnas.1418034112 PMC4413281 25847995

[ece34617-bib-0035] Johnson, C. J. , Nielsen, S. E. , Merrill, E. H. , McDonald, T. L. , & Boyce, M. S. (2006). Resource selection functions based on use – availability data: Theoretical motivation and evaluation methods. Journal of Wildlife Management, 70, 347–357. 10.2193/0022-541X(2006)70[347:RSFBOU]2.0.CO;2

[ece34617-bib-0036] Joppa, L. N. , & Pfaff, A. (2009). High and far: Biases in the location of protected areas. PLoS One, 4, e8273 10.1371/journal.pone.0008273 20011603 PMC2788247

[ece34617-bib-0037] Kendall, K. C. , Macleod, A. C. , Boyd, K. L. , Boulanger, J. , Royle, J. A. , Kasworm, W. F. , … Graves, T. A. (2015). Density, distribution, and genetic structure of grizzly bears in the Cabinet‐Yaak ecosystem. Journal of Wildlife Management, 80, 314–331. 10.1002/jwmg.1019

[ece34617-bib-0038] Kendall, K. C. , Stetz, J. B. , Boulanger, J. , Macleod, A. C. , Paetkau, D. , & White, G. C. (2009). Demography and genetic structure of a recovering grizzly bear population. Journal of Wildlife Management, 73, 3–17. 10.2193/2008-330

[ece34617-bib-0039] Kendall, K. C. , Stetz, J. B. , Roon, D. A. , Waits, L. P. , Boulanger, J. B. , & Paetkau, D. (2008). Grizzly bear density in Glacier National Park, Montana. Journal of Wildlife Management, 72, 1693–1705. 10.2193/2008-007

[ece34617-bib-0040] Lamb, C. , Mowat, G. , Gilbert, S. , McLellan, B. N. , Nielsen, S. , & Boutin, S. (2017). Density‐dependent signaling: An alternative hypothesis on the function of chemical signaling in a non‐territorial solitary carnivore. PLoS One, 12, e0184176 10.1371/journal.pone.0184176 28981540 PMC5628802

[ece34617-bib-0041] Manly, B. F. J. , McDonald, L. L. , Thomas, D. L. , McDonald, T. L. , & Erickson, W. P. (2002). Resource selection by animals: Statistical design and analysis for field studies (2nd ed., 222 pp.). Dordrecht, the Netherlands: Kluwer Academic Publishers.

[ece34617-bib-0042] Mattson, D. J. , Herrero, S. , & Merrill, T. (2005). Are black bears a factor in the restoration of North American grizzly bear populations? Ursus, 16, 11–30. 10.2192/1537-6176(2005)016[0011:ABBAFI]2.0.CO;2

[ece34617-bib-0043] McLellan, B. N. (2011). Implications of a high‐energy and low‐protein diet on the body composition, fitness, and competitive abilities of black (*Ursus americanus*) and grizzly (*Ursus arctos*) bears. Canadian Journal of Zoology, 89, 546–558. 10.1139/z11-026

[ece34617-bib-0044] Miller, S. D. (1990). Population management of bears in North America. Bears: Their Biology and Management, 8, 357–373.

[ece34617-bib-0045] Mladenoff, D. J. , & Sickley, T. A. (1998). Assessing potential gray wolf restoration in the northeastern United States: A spatial prediction of favorable habitat and potential population levels. Journal of Wildlife Management, 62, 1–10. 10.2307/3802259

[ece34617-bib-0046] Mollet, P. , Kéry, M. , Gardner, B. , Pasinelli, G. , & Royle, J. A. (2015). Estimating population size for capercaillie (*Tetrao urogallus* L.) with spatial capture‐recapture models based on genotypes from one field sample. PLoS One, 10, e0129020 10.1371/journal.pone.0129020 26087321 PMC4472805

[ece34617-bib-0047] Morehouse, A. T. , & Boyce, M. S. (2016). Grizzly bears without borders: Spatially explicit capture‐recapture in southwestern Alberta. Journal of Wildlife Management, 80, 1152–1166. 10.1002/jwmg.21104

[ece34617-bib-0048] Newmark, W. D. (1995). Extinction of mammal populations in western North American national parks. Conservation Biology, 9, 512–526. 10.1046/j.1523-1739.1995.09030512.x

[ece34617-bib-0049] Nielsen, S. E. , McDermid, G. , Stenhouse, G. B. , & Boyce, M. S. (2010). Dynamic wildlife habitat models: Seasonal foods and mortality risk predict occupancy‐abundance and habitat selection in grizzly bears. Biological Conservation, 143, 1623–1634. 10.1016/j.biocon.2010.04.007

[ece34617-bib-0050] Northrup, J. M. , Stenhouse, G. B. , & Boyce, M. S. (2012). Agricultural lands as ecological traps for grizzly bears. Animal Conservation, 15, 369–377. 10.1111/j.1469-1795.2012.00525.x

[ece34617-bib-0051] Novaro, A. J. , Funes, M. C. , & Walker, R. S. (2005). An empirical test of source‐sink dynamics induced by hunting. Journal of Applied Ecology, 42, 910–920. 10.1111/j.1365-2664.2005.01067.x

[ece34617-bib-0052] Obbard, M. E. , Howe, E. J. , & Kyle, C. J. (2010). Empirical comparison of density estimators for large carnivores. Journal of Applied Ecology, 47, 76–84. 10.1111/j.1365-2664.2009.01758.x

[ece34617-bib-0053] Paetkau, D. (2003). An empirical exploration of data quality in DNA‐based population inventories. Molecular Ecology, 12, 1375–1387. 10.1046/j.1365-294X.2003.01820.x 12755868

[ece34617-bib-0054] Paetkau, D. (2004). The optimal number of markers in genetic capture‐mark‐recapture studies. Journal of Wildlife Management, 68, 449–452. 10.2193/0022-541X(2004)068[0449:TONOMI]2.0.CO;2

[ece34617-bib-0055] Paetkau, D. , Calvert, W. , Stirling, I. , & Strobeck, C. (1995). Microsatellite analysis of population structure in Canadian polar bears. Molecular Ecology, 4, 347–354. 10.1111/j.1365-294X.1995.tb00227.x 7663752

[ece34617-bib-0056] Pettorelli, N. , Ryan, S. , Mueller, T. , Bunnefeld, N. , Jedrzejewska, B. , Lima, M. , & Kausrud, K. (2011). The Normalized Difference Vegetation Index (NDVI): Unforeseen successes in animal ecology. Climate Research, 46, 15–27. 10.3354/cr00936

[ece34617-bib-0057] Proctor, M. F. , McLellan, B. , Stenhouse, G. B. , Mowat, G. , Lamb, C. T. , & Boyce, M. S. (2018). Resource roads and grizzly bears in British Columbia and Alberta, Canada (38 pp.). Canadian Grizzly Bear Management Series, Resource Road Management, Trans‐border Grizzly Bear Project, Kaslo, BC. 10.13140/RG.2.2.11780.83846

[ece34617-bib-0058] R Development Core Team (2015). R: A language and environment for statistical computing. R Foundation for Statistical Computing Vienna, Austria http://www.R-project.org/.

[ece34617-bib-0059] Rogers, L. L. (1987). Effects of food supply and kinship on social behavior, movements, and population growth of black bears in northeastern Minnesota. Wildlife Monographs, 97, 1–72.

[ece34617-bib-0060] Royle, J. A. , Chandler, R. B. , Sollmann, R. , & Gardner, B. (2014). Spatial capture‐recapture (577 pp.). Oxford, UK: Elsevier Inc. 10.1016/C2012-0-01222-7

[ece34617-bib-0061] Royle, J. A. , & Nichols, J. D. (2003). Estimating abundance from repeated presence‐absence data or point counts. Ecology, 84, 777–790. 10.1890/0012-9658(2003)084[0777:EAFRPA]2.0.CO;2

[ece34617-bib-0062] Ruckstuhl, K. E. , & Neuhaus, P. (2002). Sexual segregation in ungulates: A comparative test of three hypotheses. Biological Reviews, 77, 77–96. 10.1017/S1464793101005814 11911375

[ece34617-bib-0063] Sawaya, M. A. , Stetz, J. B. , Clevenger, A. P. , Gibeau, M. L. , & Kalinowski, S. T. (2012). Estimating grizzly and black bear population abundance and trend in Banff National Park using noninvasive genetic sampling. PLoS One, 7, e34777 10.1371/journal.pone.0034777 22567089 PMC3342321

[ece34617-bib-0064] Sayre, N. F. , Carlisle, L. , Huntsinger, L. , Fisher, G. , & Shattuck, A. (2012). The role of rangelands in diversified farming systems: Innovations, obstacles, and opportunities in the USA. Ecology and Society, 17, 43–62. 10.5751/ES-04790-170443

[ece34617-bib-0065] Singer, F. J. , Schreier, W. , Oppenheim, J. , & Garton, E. O. (1989). Drought, fires, and large mammals: Evaluating the 1988 severe drought and large‐scale fires. BioScience, 39, 716–722. 10.2307/1311003

[ece34617-bib-0066] Stetz, J. B. , Kendall, K. C. , & Macleod, A. C. (2014). Black bear density in Glacier National Park, Montana. Wildlife Society Bulletin, 38, 60–70. 10.1002/wsb.356

[ece34617-bib-0067] Thompson, C. M. , Royle, J. A. , & Garner, J. D. (2012). A framework for inference about carnivore density from unstructured spatial sampling of scat using detector dogs. Journal of Wildlife Management, 76, 863–871. 10.1002/jwmg.317

[ece34617-bib-0068] van Manen, F. T. , Haroldson, M. A. , Bjornlie, D. D. , Ebinger, M. R. , Thompson, D. J. , Costello, C. M. , & White, G. C. (2016). Density dependence, whitebark pine, and vital rates of grizzly bears. Journal of Wildlife Management, 80, 300–313. 10.1002/jwmg.1005

[ece34617-bib-0069] Webb, N. F. , Morcos, C. , Allen, J. R. , & Frame, P. (2016). Draft management plan for black bears in Alberta. Edmonton, AB: Alberta Environment and Parks, Wildlife Management Branch.

[ece34617-bib-0070] Whittington, J. , & Sawaya, M. A. (2015). A comparison of grizzly bear demographic parameters estimated from non‐spatial and spatial open population capture‐recapture models. PLoS One, 10, e0134446 10.1371/journal.pone.0134446 26230262 PMC4521725

[ece34617-bib-0071] Williams, B. K. , Nichols, J. D. , & Conroy, M. J. (2002). Analysis and management of animal populations (817 pp.). San Diego, CA: Academic Press.

[ece34617-bib-0072] Wilson, S. M. , Madel, M. J. , Mattson, D. J. , Graham, J. M. , & Merrill, T. (2006). Landscape conditions predisposing grizzly bears to conflicts on private agricultural lands in the western USA. Biological Conservation, 130, 47–59. 10.1016/j.biocon.2005.12.001

[ece34617-bib-0073] Young, D. D. , & Beecham, J. J. (1986). Black bear habitat use at Priest Lake, Idaho. Bears: Their Biology and Management, 6, 73–80. 10.2307/3872808

