# Supplementary material for: Land tenure shapes black bear density and abundance on a multi‐use landscape
Source: Ecol Evol. 2018 Dec 18;9(1):73–89. doi: 10.1002/ece3.4617 (PMC6342132; doi:10.1002/ece3.4617)
Supplement: Supplementary file 1 [file ECE3-9-73-s001.docx]

# Supporting Material

August 4, 2018

Citation: Loosen, A., A. Morehouse, and M. Boyce. 2017. Land Tenure Shapes Black Bear Density and Abundance on a Multi-Use Landscape. Ecology and Evolution.

Additional supporting material may be found in the online version of this article at the publisher’s web-site. Included in this section are detailed genetic results, candidate resource selection function (RSF) models, RSF values comparing rub objects to random locations, and distribution of sex-specific black bear RSF values.

## Detailed Genetic Results

In 2013, we visited 855 rub objects and 49 opportunistic grids. We detected black bears at 52% of the traps (*n* = 466). We submitted 4,554 hair samples (4,179 rub object; 375 opportunistic) to WGI for analysis in 2013. Genetic results were derived from two data sets. The first data set (genetic data) included individuals identified to be black bears during the single-locus pre-screen of data (G10J; 54.4%). Samples that were assigned a high-confidence G10J score had an 83% (*n* = 564) genotyping success rate, 16% (*n* = 108) of samples failed at >3 loci and were excluded from further analysis, and 1% (*n* = 4) had genetic material from >1 individual. The second data set (visual data set) included samples identified as black bear during visual inspection (i.e., jet black) before genotyping. Samples had a 74% (*n* = 67) genotyping success rate and 26% (*n* = 23) of samplings failed at >3 loci. Data from the visual and genetic data sets were combined and resulted in 306 detections of 126 males and 177 detections of 101 female black bears over 8 occasions in 2013. For males, we had 160 recaptures of 126 individuals and 58 recaptures of 101 individual females.

In 2014, we visited 873 rub objects and 54 opportunistic grids. We detected black bears at 48% of the traps (*n* = 444). We submitted 3,912 hair samples (3,597 rub object and 315 opportunistic) for analysis in 2014. Like 2013, samples visually identified as black bear were restricted to the visual data set. These samples had a 68% (*n* = 111) genotyping success rate, 32% (*n* = 52) failed at >3 loci, and 1% (*n* = 1) had genetic material from >1 individual. Unlike 2014, samples with odd-numbered alleles at G10J were not set aside and WGI staff conducted a simultaneous analysis of grizzly and black bear samples, using the 8-locus first pass as a quality control measure. These samples had a 96% (*n* = 494) genotyping success rate and 4% (*n* = 21) failed at >3 loci. Combined, this resulted in 294 detections of 122 males and 168 detections of 100 females in 2014. For males, we had 160 recaptures of 122 individual males and 53 recaptures of 100 females. Across both years, 1,236 samples were assigned individual multi-locus genotypes to 347 black bears (186 males, 161 females). Of these, 107 individuals were detected in both years.

Table S1. Resource selection function (RSF) candidate models for male and female black bears in southwestern Alberta, Canada. Please see methods for descriptions of covariates.

| Model number | Description |
| --- | --- |
| 1 | water + tertiary rd + canopy + tenure |
| 2 | burn + NDVI + shrub + tenure + GBU |
| 3 | TRI + primary rd + building + canopy |
| 4 | TRI + secondary rd + building + canopy |
| 5 | water + burn + canopy + tertiary rd |
| 6 | elevation + primary rd + building + canopy |
| 7 | elevation + primary rd + deciduous + tenure |
| 8 | burn + tertiary rd + shrub + building |
| 9 | burn + primary rd + deciduous + building |
| 10 | burn + primary rd + agriculture + building |
| 11 | ~1 |

Figure S1. Resource selection function (RSF) values in the area of inference in southwestern Alberta (2013–2014), comparing habitat covariates associated with all rub object locations to random locations. The area of inference excludes the white area within the study area boundary. We used Waterton Lakes National Park as the reference area to calculate abundance and density.
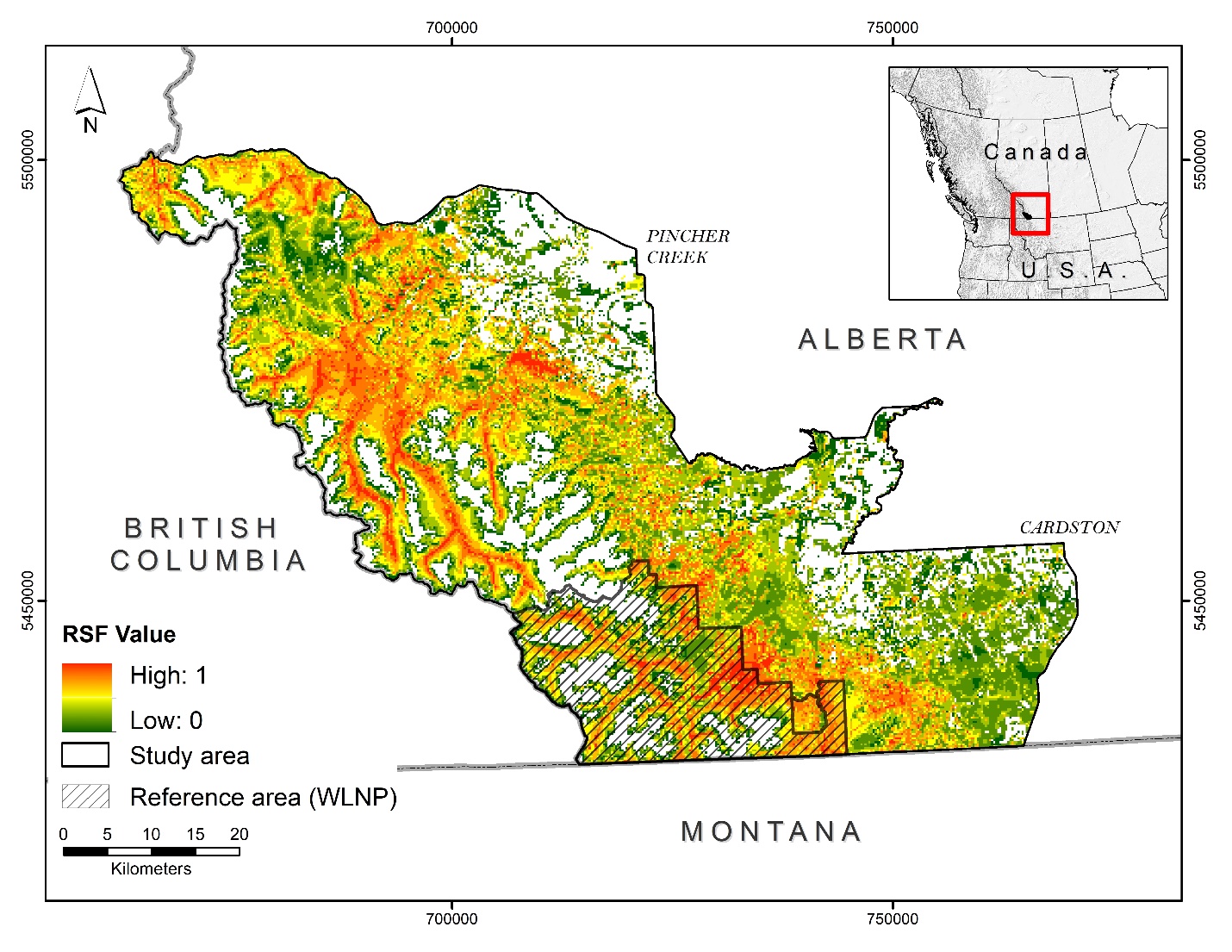


Figure S2. Distribution of sex-specific black bear resource selection (RSF) function values in the reference (protected) area, private lands, and Crown lands in southwestern Alberta, Canada (2013–2014).
